# Metastatic Spread in Prostate Cancer Patients Influencing Radiotherapy Response

**DOI:** 10.3389/fonc.2020.627379

**Published:** 2021-03-04

**Authors:** Daria Klusa, Fabian Lohaus, Giulia Furesi, Martina Rauner, Martina Benešová, Mechthild Krause, Ina Kurth, Claudia Peitzsch

**Affiliations:** ^1^ National Center for Tumor Diseases (NCT), Dresden, Germany; ^2^ German Cancer Research Center (DKFZ), Heidelberg, Germany; ^3^ Faculty of Medicine and University Hospital Carl Gustav Carus, Technische Universität Dresden, Dresden, Germany; ^4^ Helmholtz-Zentrum Dresden—Rossendorf (HZDR), Dresden, Germany; ^5^ Department of Radiotherapy and Radiation Oncology, Faculty of Medicine and University Hospital Carl Gustav Carus, Technische Universität Dresden, Dresden, Germany

**Keywords:** prostate cancer, radiotherapy, metastasis, circulating tumor cells, radiopharmacy

## Abstract

Radiotherapy and surgery are curative treatment options for localized prostate cancer (PCa) with a 5-year survival rate of nearly 100%. Once PCa cells spread into distant organs, such as bone, the overall survival rate of patients drops dramatically. The metastatic cascade and organotropism of PCa cells are regulated by different cellular subtypes, organ microenvironment, and their interactions. This cross-talk leads to pre-metastatic niche formation that releases chemo-attractive factors enforcing the formation of distant metastasis. Biological characteristics of PCa metastasis impacting on metastatic sites, burden, and latency is of clinical relevance. Therefore, the implementation of modern hybrid imaging technologies into clinical routine increased the sensitivity to detect metastases at earlier stages. This enlarged the number of PCa patients diagnosed with a limited number of metastases, summarized as oligometastatic disease. These patients can be treated with androgen deprivation in combination with local-ablative radiotherapy or radiopharmaceuticals directed to metastatic sites. Unfortunately, the number of patients with disease recurrence is high due to the enormous heterogeneity within the oligometastatic patient population and the lack of available biomarkers with predictive potential for metastasis-directed radiotherapy. Another, so far unmet clinical need is the diagnosis of minimal residual disease before onset of clinical manifestation and/or early relapse after initial therapy. Here, monitoring of circulating and disseminating tumor cells in PCa patients during the course of radiotherapy may give us novel insight into how metastatic spread is influenced by radiotherapy and vice versa. In summary, this review critically compares current clinical concepts for metastatic PCa patients and discuss the implementation of recent preclinical findings improving our understanding of metastatic dissemination and radiotherapy resistance into standard of care.

## Introduction 

Standard of care for metastatic prostate cancer (PCa) patients is systemic therapy, e.g. androgen deprivation therapy (ADT) or docetaxel-based chemotherapy. First-line therapy for non-metastatic, castration resistant prostate cancer (CRPC) patients is systemic ADT based on second-generation nonsteroidal antiandrogens enzalutamide or apalutamide with a significant benefit in metastasis free survival. At prostate-specific antigen (PSA) recurrence after definitive local therapy, e.g. radical prostatectomy, radiotherapy, or both, prostate-specific membrane antigen-based imaging can identify local recurrence or oligo-metastases (1). This increases the number of diagnosed patients with asymptomatic metastasis and rising PSA level. High-dose external beam radiotherapy can successfully control those lesions in hormone-naïve and even in metastatic CRPC patients (2–4). However, up to 70% of these patients will experience further disease progression. Established methods for stratification of PCa patients into prognostic subgroups are solely based on PSA kinetics (e.g. PSA velocity, PSA doubling time), but not on biological, disease-related differences. Whether the observed differences in response are related to specific biological phenotypes is often hypothesized, but not clinically proven yet. Therefore, the characterization of cellular signatures for radiotherapy response coming from the primary tumor or distant metastasis, e.g. based on liquid biopsy analysis, has the potential to detect underlying resistance and metastasis-initiating mechanisms. Despite the increasing understanding of the cellular and molecular processes underlying the metastatic cascade, there are still key questions to answer: How do metastases differ molecularly and phenotypically from the primary tumor? Is it possible to predict metastatic spread from signatures within the primary tumor? Can the cellular composition and degree of heterogeneity in the metastases be used as signature for patient stratification? How efficient can metastasis-directed therapy be implemented into clinical routine and do PCa patients benefit? To answer the raised questions, this review summarizes the current knowledge about the metastatic cascade in PCa, introduces state-of-the-art imaging modalities to visualize microscopic metastatic lesions, and discusses novel developments in the field of metastasis-directed therapies. Moreover, we introduce the concept of circulating and disseminating tumor cells and discuss their prognostic potential for patient stratification and therapy monitoring.

## Characteristics of Metastatic Sites in Prostate Cancer

### Routes of Metastasis in Prostate Cancer

**Figure 1:**
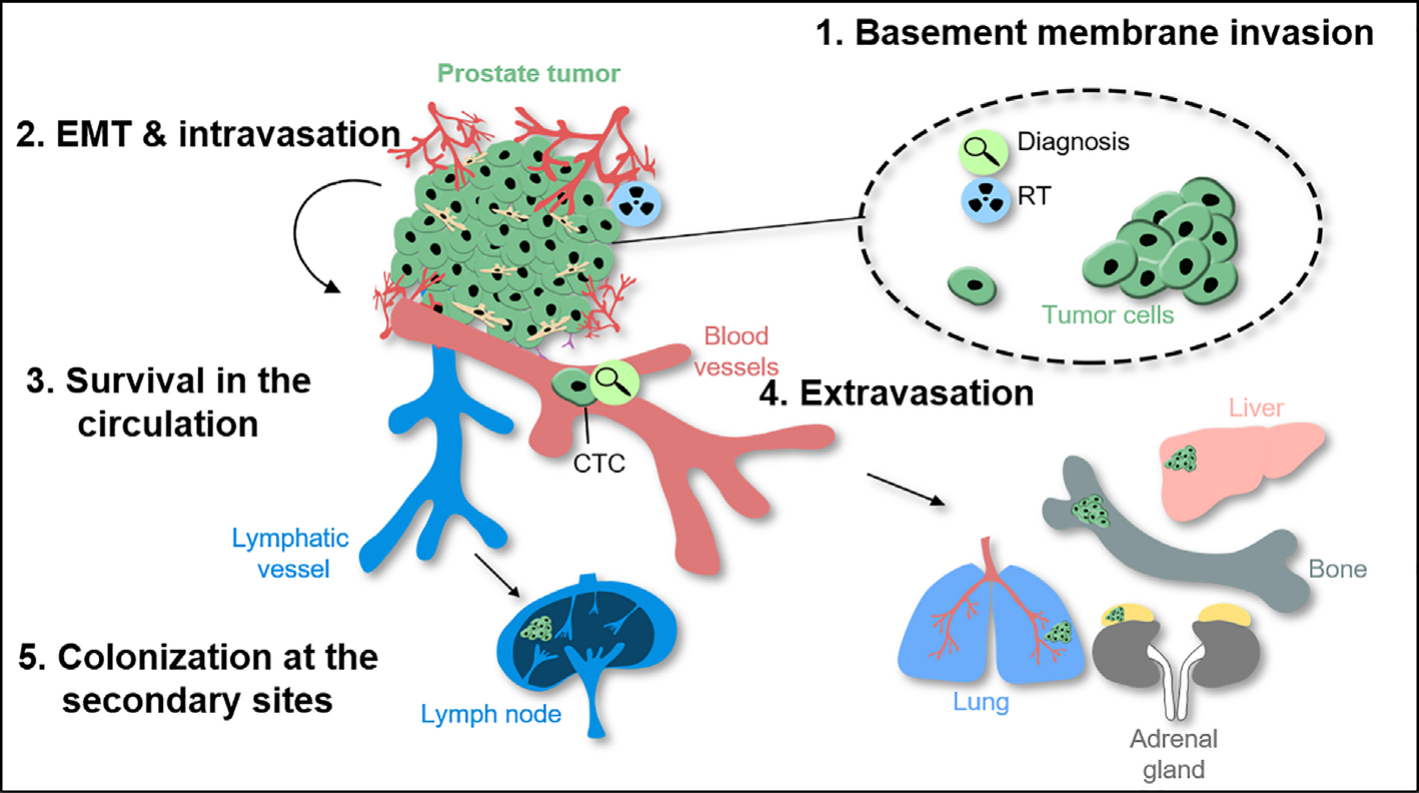
The metastatic cascade in prostate cancer and molecular effects of radiotherapy. During tumor invasion throughout the basement membrane and further migration into surrounding normal tissue, prostate cancer (PCa) cells use epithelial-to-mesenchymal-transition (EMT) as biological program. Intravasation allows tumor cells to enter the circulation including the lymphatic and/or vascular system. To extravasate into distant tissue prostate circulating tumor cells (CTCs) have to attach to the inner vessel wall before leaving the blood system. Once the cells left the circulation they may settle down and colonize secondary organs e.g. within bones as the main metastatic site for PCa patients.

The invasion of tumor cells into the surrounding tissue and the seeding of metastases remains a challenging issue, as it represents the main cause of increased mortality among patients (5, 6). During metastasis formation, tumor cells undergo a complex multi-stage intra- and intercellular remodeling process. The metastatic cascade can be described by five major steps: 1) invasion throughout the basement membrane and migration into the surrounding tissue; 2) intravasation into the vasculature or lymphatic system; 3) survival within the circulation; 4) extravasation from the vasculature into the tissue; and 5) colonization and formation of metastatic lesions at secondary sites (**Figure 1**) (5, 7). Each stage represents enormous environmental pressure and energetically demanding conditions for the cancer cells. The whole process is thought to be extremely inefficient and less than 0.1% of the cancer cells that detach from the primary tumor survive within 24 h (8, 9). Moreover, different tumor entities display a different metastatic pattern depending on cell-intrinsic and extrinsic regulatory mechanisms. The so-called pre-metastatic niches support the adaptation of cancer cells to their new environment and increase the rate of metastases. Despite the circulation of tumor cells is a random process, the metastasis formation follows specific routes. This was already proposed within the seed-and-soil theory by Sir Stephen Paget in 1889 who stated that distant organs provide a specific environment as soil for cancer cells to seed secondary tumors (10). The concept of metastatic organotropism defines tumor entity-specific target organs. Organotropism is regulated by circulation pattern, tumor cell-intrinsic signaling, organ-specific niches, and the communication between tumor cells and the host microenvironment (11). PCa cells preferentially metastasize into bone and lung as secondary site. Within a large autopsy study of 19,000 cancer patients including 1,600 PCa patients, the bone was with 90% the most frequent metastatic site in PCa (12). This was followed by metastasis to the lungs (46%), liver (25%), pleura (21%), and adrenals (13%). Within the bone, metastases were mostly detected at the spine (90%), whereas ribs (18%), long bones (15%), and skull (8%) were less frequently affected. Within the spine, the lumbar spine is affected most (90%), followed by the thoracic (66%) and cervical spine (38%), suggesting that PCa cells follow a venous spread from the prostate to the spine. Besides the hematogenic spread through the blood stream, cancer cells can enter the lymphatic system. As such, PCa cells favor settlement into the paraaortic, pelvic, and mediastinal lymph nodes (12). Of note, there is a strong association between lymphatic and hematogenous spread. Over 84% of the tumors with paraaortic and pelvic lymphatic metastasis also displayed hematogenous metastasis, whereas when paraaortic and pelvic metastasis were absent, only 16% showed hematogenous spread. Finally, the nodal status correlates strongly with the occurrence of distant metastases, and both of them are associated with advanced histological grade and tumor growth, highlighting the importance of the detection of metastasis as a major prognostic factor in PCa. The occurrence of lymph node metastasis in patients with PCa indicates a poor prognosis (13–17) and it is frequently associated with a poor response to radical prostatectomy and radiation therapy. Thus, it is critical to understand the mechanisms underlying lymph node metastasis to improve the care of patients with PCa.

### Characteristics of Lymph Node Metastasis

**Figure 2:**
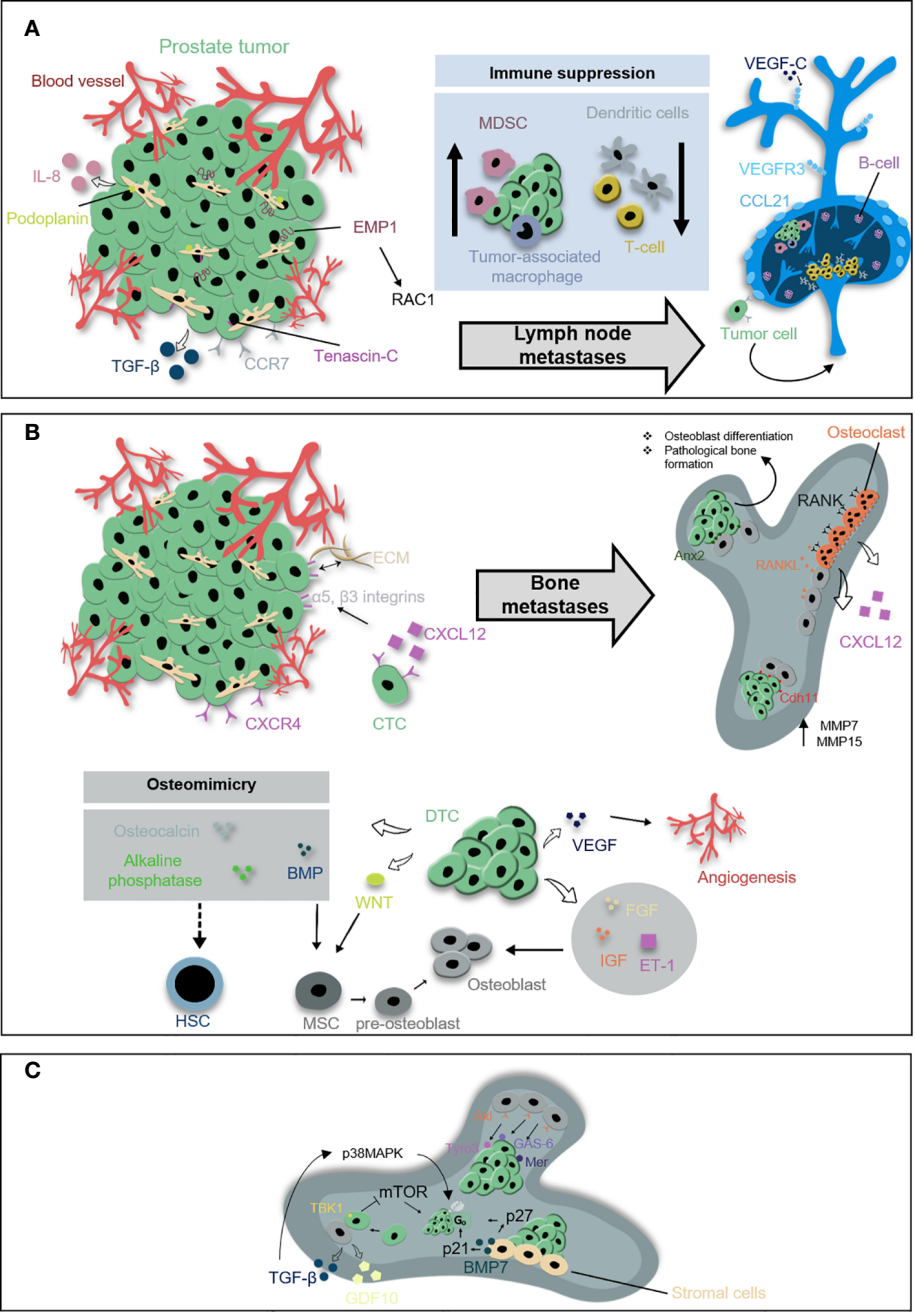
Prostate metastases within lymph nodes and bone. **(A)** Prostate cancer cells form a pre-metastatic niche in lymph nodes prior dissemination and colonization to the lymph nodes. The decreased immune function is reflected by the reduced density of dendritic cells and T cells but also by the attraction of myeloid-derived suppressor cells (MDSCs) or tumor-associated macrophages. PCa cells and surrounded cancer-associated fibroblasts release soluble factors such as tumor necrosis factor α (TNF-α), CC-chemokine ligand 21 (CCL21), and interleukin-8 (IL-8) involved in pre-metastatic niche formation within lymph nodes. CCL21 induces chemokine receptor 7 (CCR7) on PCa cells. Epithelial membrane protein 1 (EMP1) is induced in PCa cells after contact with prostate stromal cells and likely promotes metastasis into the lymph nodes *via* a Rac1-dependent mechanism. Lymph-angiogenesis involves the outgrowth and remodeling of lymphatic vessels and is induced by vascular endothelial growth factor C (VEGF-C) secreted from PCa cells and vascular endothelial growth factor receptor 3 (VEGFR3) on lymphatic vessels. **(B)** Beside lymph nodes, the bone is a major metastatic site for PCa. The C-X-C motif chemokine ligand 12 C-X-C chemokine receptor type 4 (CXCL12-CXCR4) signaling guides disseminating PCa cells into the bone where they colonize within already formed pre-metastatic endosteal niche close to osteoblasts. CXCL12/CXCR4 binding enhances the expression of α5 and β3 integrins in PCa cells and reinforces their adhesion to the extracellular matrix (ECM). Prostate disseminated tumor cells (DTCs) target the endosteal niches and compete with hematopoietic stem cells (HSCs) in order to survive. In the niche, DTCs release factors originally involved in bone formation and maintenance, such as osteocalcin, alkaline phosphatase, and bone morphogenetic proteins (BMP). DTCs support osteoblastic activity through the release of fibroblast growth factors (FGFs), insulin-like growth factors (IGFs), VEGFs, endothelin 1 (ET-1), Wnt pathway-related factors, and BMPs. Moreover, adhesion proteins facilitate the metastatic spread to the bone, including cadherin-11 (Cdh11) upregulating metalloproteinases MMP-7 and MMP-15. Osteoblasts redirect prostate cancer cells toward the endosteal niche by expressing Annexin2 (Anx2). PCa cells and other cells within the bone microenvironment subsequently are co-regulated throughout a vicious cycle e.g., *via* receptor activator of nuclear factor kappa-B ligand (RANKL). **(C)** Tumor cells within a quiescent phase, also known as dormancy, exhibit a reversible cell cycle arrest in G0 phase. Stroma-derived growth arrest-specific protein 6 (GAS-6) induces dormancy by binding the Tyro3, Axl, and Mer receptor tyrosine kinases. Dormancy is also regulated by the expression of TANK-binding kinase 1 (TBK1) induced by osteoblast and PCa cell interactions inhibit mTOR signaling and induce G0 phase. Stroma-derived BMP-7 suppress the proliferation of PCa cells through increased expression of the mitotic inhibitors p21 and p27. Additional regulators of dormancy are GDF10 and TGF-β which phosphorylates p38MAPK.

Lymph node metastasis positive PCa patients are at high risk for further disease progression (13–17) and a poor response to radical prostatectomy and radiation therapy. However, data from randomized clinical studies demonstrated that local therapy in combination with ADT can result in long-term disease control (18, 19). Thus, it is critical to understand the mechanisms underlying lymph node metastasis to improve the care of patients with PCa. PCa cells form a pre-metastatic niche in lymph nodes as tumor-adjacent lymph nodes display changes in the architecture and immune function even before tumor cell dissemination and lymph node colonization. The decreased immune function is reflected by the reduced density of paracortical antigen-presenting dendritic cells and T cells (20, 21), but also by the attraction of immune-suppressive cell types such as myeloid-derived suppressor cells or tumor-associated macrophages (22). This is a critical step to escape recognition and elimination by immune cells in the lymph nodes. Several means of bi-directional pre-metastatic niche communication have been proposed, e.g. that the lymphatics produce factors that attract PCa cells, but also that PCa cells or other cells present in the tumor microenvironment, such as cancer-associated fibroblasts (CAFs), produce growth factors and cytokines that promote lymph-angiogenesis. Recently, the CC-chemokine ligand 21-CC chemokine receptor 7 (CCL21-CCR7) axis has been implicated in PCa migration into the lymph nodes (23). High expression of CCL21 was detected in lymph node metastasis of PCa patients. The tumor necrosis factor α (TNF-α) has been shown to induce CCR7, the receptor for CCL21, and migration of PCa cells. Moreover, the epithelial membrane protein 1 (EMP1) was identified to be induced in PCa cells after contact with stroma cells subsequently promoting cancer progression and metastasis formation in the lymph nodes and lung *via* a Rac1-dependent mechanism (24). These tumor-stroma interactions are facilitated by the glycoprotein podoplanin and the extracellular matrix protein tenascin-C expressed by CAFs. A high podoplanin and tenascin-C expression in the stroma of PCa biopsies strongly correlates with tumor stage, lymph node metastasis, and poor prognosis (25, 26). Lymph-angiogenesis studies identified the vascular endothelial growth factor receptor 3 (VEGFR3) and its ligands vascular endothelial growth factor (VEGF) -C and -D as critical determinants of lymphatic endothelial cell proliferation and sprouting of lymphatic vessels. In PCa, expression of VEGF-C and VEGFR3 is highly correlated with regional lymph node metastasis and associated with a poor prognosis (27–29). A recent study showed that blocking VEGF-C or VEGFR3 with antibodies or RNA interference reduced lymph node and distant metastasis, while not interfering with the growth of the primary tumor (30). This is in contrast to VEGFR2, whose inhibition reduced metastasis mainly due to the reduction of primary tumor growth by suppressed angiogenesis. Recently, phase I/II clinical trials have been completed to test the safety of VEGFR3 or VEGFR2 inhibition in patients with advanced solid tumors. Despite good tolerability, VEGFR3 or VEGFR2 inhibition showed no benefit in suppressing tumor growth or lymph node metastasis. However, these studies show that VEFGR inhibition is safe paving the way for potential combination therapies (31, 32) (**Figure 2A**).

Taken together, the concept of the pre-metastatic niche also holds true in prostate cancer lymph node metastasis. Identifying key pathways of niche communication may have significant implications for prognostic and therapeutic purposes in prostate cancer, such as targeting the VEGR3-VEGF-C axis to halt the progression of lymph node metastasis and improve the patient´s prognosis.

### Characteristics of Bone Metastasis

The propensity of PCa cells to metastasize to the skeleton, and further progression to other organs, is a principal cause of morbidity and mortality among the male population. Although bone metastases can be initially asymptomatic, their consequences are often detrimental due to the occurrence of skeletal-related events such as fractures, bone pain, and spinal cord compression that markedly reduce the quality of life. While most of the solid tumors, such as breast cancer and melanoma, tend to cause osteolytic lesions with excessive bone resorption, bone lesions resulting from PCa are primarily osteoblastic and associated with uncontrolled low-quality bone formation (33).

Similar to lymph node metastasis, one of the crucial steps in the establishment of bone metastases is the formation of the metastatic niche (34). This process relies on the interactions between prostate cancer cells and bone resident cells to create a pro-tumorigenic environment in an otherwise non-permissive site. During the initial phase of bone metastasis, prostate cancer cells target the endosteal niches and compete with hematopoietic stem cells in order to survive and thrive (35). Once in the niche, disseminated prostate cancer cells invade the surrounding tissue by acquiring a bone-like phenotype, also known as osteo-mimicry. In fact, tumor cells modify their molecular signature by releasing factors originally involved in bone formation and maintenance, such as osteocalcin, alkaline phosphatase, and bone morphogenetic proteins (36, 37). This leads to the disruption of physiological bone remodeling and the onset of pathological lesions.

Among all the molecules that actively participate in PCa metastasis, bone-derived-chemokines have been shown to be crucial for a successful colonization of the skeleton. One of the most studied chemokines secreted by bone marrow stromal cells and mature osteoblasts is the C-X-C motif chemokine ligand 12 (CXCL12). Experimental evidence revealed that secretion of osteoblastic CXCL12 triggers dissemination of tumor cells from the bloodstream to the target site by binding the receptor C-X-C chemokine receptor type 4 (CXCR4) located on the tumor cells (38, 39). Inhibition of CXCL12/CXCR4 axis using a CXCR4 antagonist compromised tumor growth by altering the interaction of cancer cells with osteoblast niches (40, 41). However, this treatment failed to reduce already established metastasis (41, 42), suggesting that CXCL12/CXCR4 axis is relevant during the initial colonization phase, but not at the late stage of the disease. In addition, it has been shown that the binding of CXCL12 to its receptor enhances the expression of α5 and β3 integrins in PCa cells, two major glycoproteins involved in tumor progression (43).

Other factors involved in tumor retention within the bone marrow are the adhesion proteins. Huang et al. demonstrated that the expression of cadherin-11 in PCa cells enhances the metastatic spread to bone by providing a physical link to the osteoblastic component (44). In accordance with that, clinical specimens confirmed higher levels of cadherin-11 in metastasis compared to the primary site (45). In addition, gene expression analyses showed that cadherin-11 facilitates PCa migration and invasion through upregulation of invasive-related genes, such as metalloproteinases (MMP) -7 and -15 (44). Results from studies investigating the role of bone cells for prostate carcinogenesis further revealed that osteoblasts redirect PCa cells toward the endosteal niche by expressing annexin 2, an adhesion molecule involved in osteoclast activation and mineralization (46, 47). Interaction of tumor cells with osteoblasts activates gap junction signaling with a subsequent impairment of the bone matrix structure (48). For example, high expression of the gap junction subunit connexin 43 has been reported to alter osteoblast cytoskeletal organization and enhance migration of tumor cells (49) (**Figure 2B**).

After colonization to the bone, PCa cells adapt to the foreign microenvironment and escape immune surveillance by entering a quiescent phase, also known as dormancy. Dormant tumor cells exhibit a reversible cell cycle arrest in G0-G1 phase, in which they remain viable but do not proliferate. Thus, quiescent cancer cells represent a clinical challenge since they are commonly chemo-resistant. Stroma-derived growth arrest-specific protein 6 (Gas6) has been shown to induce dormancy in PCa cells by binding to the receptor tyrosine kinases family member Tyro3, Axl, and Mer (TAM) and downstream activation of multiple signaling pathways, including MAPK and phosphoinositide 3-kinase (PI3K)-Akt (50). The engagement of annexin 2 on PCa cells stimulate Axl, which contributes to a dormant state and drug resistance in metastatic cells (51). While Axl levels are significantly high in quiescent cells, Tyro3 has been associated with rapid tumor growth, suggesting that a balance between the expression of Axl and Tyro3 might influence the switch of PCa cells from a dormant to proliferative state and vice versa (52). Moreover, Kim et al. found that the binding of PCa cells to osteoblasts in the endosteal niche induces the expression of TANK-binding kinase 1 (TBK1) in tumor cells, which in turn inhibits mTOR signaling pathway and induces cell cycle arrest (53). Finally, recent studies showed that two members of the transforming growth factor beta (TGF-β) superfamily, TGF-β2, and BMP-7, play a crucial role in metastatic dormancy. Specifically, osteoblast-derived TGF-β2 activates TGF-βRIII signaling in PCa cells with a subsequent phosphorylation of p38MAPK and interruption of the cell-cycle in G1-phase through the increase of the cell cycle inhibitor p27 (54). Similarly, stroma-derived BMP-7 suppresses the proliferation of prostate cancer cells through an increased expression of the mitotic inhibitors p21 and p27 (**Figure 2C**). Even though dormancy ensures tumor cell survival within the bone, the formation of detectable metastasis requires the exit of PCa cells from the quiescent state. Reactivation can be achieved by endosteal niche remodeling due to activation of osteoclastogenesis, meaning the differentiation of bone-resorbing osteoclasts from myeloid precursor cells (55). For instance, *in vivo* experiments have shown that induced by castration bone resorption leads to increased bone metastasis, a process that can be prevented using osteoclastic inhibitors, such as bisphosphonates or receptor activator of nuclear factor kappa-B ligand (RANKL) inhibitors (56). Uncontrolled activation of osteoclasts promotes a vicious cycle of growth factor signaling between bone resident cells and cancer cells leading to a final outgrowth of the tumor. From a clinical perspective, several trials have investigated the efficacy of osteoprotective drugs in advanced PCa (57). Administration of bisphosphonates, e.g., zoledronic acid, has consistently shown protection against bone loss in patients receiving endocrine therapy compared with placebo (58, 59). Despite promising results obtained using animal models (56), there is no clear evidence of survival improvement in humans (60, 61). Besides zoledronic acid, denosumab, a RANKL inhibitor has been validated as an effective antiresorptive agent in the treatment of bone metastasis in PCa patients. In a randomized phase III study, denosumab significantly reduced skeletal-related events and improved pain control compared to bisphosphonates (62). However, more long-term follow-up studies are needed to identify potential complications and define the time point for treatment initiation (63, 64).

In summary, despite significant progress into mechanisms of PCa, further analyses need to be addressed in order to unravel the molecular basis of bone metastasis at both early and late stages. This will help to reduce the rate of metastasis formation and eventually develop new molecular targeting strategies for PCa management.

### Molecular Characteristics of Metastasis in Comparison to Primary Tumor

The complex metastatic cascade is accompanied by a multitude of molecular and phenotypic changes within tumor cells to enable metastasis formation. When cancer cells leave the primary tumor, cell-autonomous characteristics that promote survival in the circulation and within target organs are extremely important (7). Genetic and epigenetic alterations within the primary tumor and acquired at the metastatic site contribute to phenotypic changes and corresponding host interactions (65). A genetic relationship between the primary tumor and the metastases is rather seen as linear progression whereas genetic divergence is interpreted as parallel development (66). Within a published study in 2009, Li et al. examined copy number variations (CNVs) of multiple metastases within 24 patients and found that a majority of samples had the same CNVs in primary tumor and metastases pointing to a linear progression model with monoclonal origin for metastatic PCa (66, 67). A 17-year longitudinal sampling of lethal PCa cases with subsequent comprehensive genomic and pathologic analysis supported this finding. Haffner et al. traced the lethal metastatic clone back to the specific lesion of origin (68). Surprisingly, the lethal clone, defined by the presence of phosphatase and tensin homolog (*PTEN)*, tumor protein P53 (*TP53*), and speckle-type POZ protein *(SPOP*) mutations arose from a tumor region with pathological characteristics of a low-risk area and low Gleason score (68, 69). Primary PCa displays an enormous heterogeneity, which is reflected by distinct molecular subtypes and a wide variety of clinical outcomes (70). A comprehensive molecular analysis of 333 primary PCa samples from The Cancer Genome Atlas (TCGA) defined seven subtypes based on erythroblast transformation specific transcription factors (*ETS*) fusions or mutations in *SPOP*, forkhead box A1 (*FOXA1*), and isocitrate dehydrogenase 1 (*IDH1*), but demonstrated a substantial epigenetic heterogeneity within the subgroups (70). When comparing sequencing data from primary PCa and metastatic CRPC, it becomes clear that metastases carry significantly more mutations and copy number alterations than primary tumors (65, 71). In particular, metastases show frequent alterations of the androgen receptor (*AR*), *TP53*, retinoblastoma-associated protein (*RB1*), lysine N-methyltransferase *KMT2C* and *KMT2D*, DNA repair genes, and members of the phosphoinositide 3-kinases (PI3K) signaling pathway (71). A hallmark of PCa is the dependency on AR signaling pathways for tumor progression illustrated by the increased abundance of AR amplification. Prospective AR diagnostics impact on the clinical choice for AR-specific targeting therapies (71). In a multicenter study, a significantly higher incidence of germline mutations was found in metastatic PCa patients (11.8%) compared to 4.6% in men with localized PCa (72). Mutations were found in 16 genes, including key regulators of DNA-repair such as *BRCA2*, *ATM*, *CHEK2*, *BRCA1*, *RAD51D*, and *PALB2*. These defects in DNA repair may contribute to a further increase of mutational burden. Moreover, they can be accounted as metastasis driver mutations impacting clonal expansion while passenger mutations have no effect on the cancer cell (73). Within the primary tumor, specific genes are selectively mutated at early or later stages during tumor progression enforcing clonal evolution (74).

### Clonal Evolution During Metastatic Cascade

Major determinants for metastasis formation are tumor cell adaptability and plasticity to its changing microenvironment during disease progression and therapeutic intervention (75). Early metastatic features are already selected within the primary tumor under immune pressure, within hypoxic areas or at the invasive front (7). In PCa, it appears that individual clones within the primary tumor acquired pro-metastatic properties and the most potent clones are responsible for metastasis formation or re-seeding of the primary tumor-bed e.g., after surgical removal (7, 76). Therefore, PCa cells undergo an epithelial-to-mesenchymal transition (EMT) in response to TGF-β secreted by surrounding stromal cells. EMT is a reversible phenotypic switch where epithelial cancer cells lose their intercellular adhesion and polarization in order to gain motility and invasiveness (77).

Clonal evolution analysis in metastatic PCa patients based on a deep sequencing technique revealed a branching phylogenetic architecture from primary tumor to distant metastasis with stage-specific mutational signatures (76). Interestingly, Hong et al. detected clones from various tumor stages within the blood implying multiple, temporally separated waves of tumor cell dissemination from the primary tumor. This parallel model of prostate metastasis assumes that metastasis-initiating clones may occur already before clinical diagnosis of the primary tumor (65). Another study found that an initial hormone-naïve metastasis clone contained two sub-populations after treatment. One subclone derived from the original clone and the other origined from distant sacral metastasis. This points to the requirement of specific genetic alterations for metastatic colonization that may evolve outside of the primary tumor and describes, for the first time, a pre-requisite for the parallel progression model (65, 76). For example, the acquisition of *TP53* missense mutations in low-frequency sub-clones inside the primary tumor and their subsequent accumulation in metastasis samples may indicate that *TP53* mutations increase the metastatic potential of tumor clones and are key drivers for PCa metastasis (74, 76). In ten patients with metastatic CRPC, Gundem et al. found evidence for the existence of polyclonal seeding at distant sites. They found that metastases frequently spread from metastasis to metastasis, either by de-novo monoclonal seeding of daughter metastases or through the transfer of multiple tumor clones (5/10 patients, 50%). Within those lesions, they found mutations in tumor suppressor genes occurring as a single event in distinct clones, whereas mutations in AR signaling were detected simultaneously in multiple metastatic clones (78). Additional studies validated this polyclonal seeding based on the genomics analysis (65, 78), which indicates that subclones may cooperate or compete at all steps during metastatic cascade (78). Another study published by Gundem et al. investigated the polyclonal seeding under therapeutical pressure and identified oncogenic alterations associated with ADT resistance such as *MYC* amplification or *CTNNB1* mutation. The authors hypothesize that polyclonal expansion may be driven by distinct resistance mechanisms (78, 79). They also found that multiple metastases were more closely related to each other than to the primary tumor. Phylogenetic trees illustrate the acquisition of mutations in PCa metastases either linear, parallel, or branched (78). It seems that metastatic PCa cells share a common genetic fingerprint and thus may share a common heritage.

To sum up the molecular part, *ETS* fusion and mutations in *FOXA1*, *FLI1*, *SPOP*, and *IDH1* are tumorigenic drivers and the basis for PCa heterogeneity (70). Missense mutations of *TP53* and *PTEN* occur before or at early stages during metastatic cascade (68, 76) determining them as metastasis drivers. One interesting finding is that AR expression, which is altered in >60% of metastatic prostate cancer (80), changes after the occurrence of metastases. Currently, it is unclear whether rare subclones originate from the primary tumor or early metastases harbor AR alterations and promote ADT resistance. It may be also possible that such alterations occur after metastasis formation and ADT (81). Finally, it has been demonstrated that metastatic spread is not unidirectional and metastatic clones may re-seed the original tumor bed (76, 78). This impacts the clinical characteristics of metastatic PCa and therapeutic options.

## Diagnostic Imaging of Prostate Cancer Patients With Disseminated Disease

### Imaging of Metastasis Status in Prostate Cancer Patients

The screening for PSA level in the serum of patients was introduced in the late 1980s (82) and enabled a dramatic increase in early PCa detection (83). On the other hand, PSA is not solely a PCa-specific biomarker and, as such, leads to overdiagnosis and overtreatment of clinically insignificant cases, representing a significant burden for patients (84). Moreover, absolute PSA level does not always correlate with prognosis (85). Therefore, more specific and sensitive PSA-based values like PSA density (PSAD) (86), PSA velocity (PSAV) (87), free-to-total PSA (F/T PSA) (88), and PSA doubling time (PSADT) (89) are seen as options with stronger predictive value. For example, PSADT is defined as the length of time for two-fold PSA level increase. A PSADT <6 months is strongly associated with metastatic disease, increased PCa mortality (90), and relapse (91). Nonetheless, the reported benefit of PSADT in PCa management did not enter clinical routine and some studies even reported discrepant results indicating that further studies are required to determine the reliability of PSADT and other available biomarkers (92–94).

Recommended diagnostics for men at risk of extra-prostatic cancer spread include computer tomography (CT), skeletal scintigraphy and positron emission tomography (PET) as well as combined imaging modalities like single photon emission computed tomography (SPECT)/CT, PET/CT, and PET/magnetic resonance imaging (MRI). The most promising strategy is represented by radiotracer-based PET imaging which mainly employs changed metabolic activity or specifically overexpressed receptors (95). The choice of a respective radiotracer has to be considered carefully as one single radiotracer is usually not suitable to visualize all clinical stages of PCa. Moreover, its utilization is strongly dependent on the level of malignant tissue, tumor heterogeneity (96), and previously applied treatments (97). The 2-deoxy-2-^18^F-fluoro-D-glucose (^18^F-FDG) is the most commonly used radiotracer in clinical PET imaging worldwide. It is seen as limited with rather low overall sensitivity for PCa compared to other malignancies with a higher glycolytic rate (98). In contrast, patients with discordant ^18^F-FDG-avid metastatic CRPC are usually identified with a poor prognosis and short overall survival (99). Thus, ^18^F-FDG-PET imaging represents a relevant prognostic indicator correlating with enhanced glucose transporter 1 expression in high-risk PCa patients (100). The androgen receptor (AR) represents a key molecular target for AR-binding 16β-^18^F-fluoro-5α-dihydrotestosterone (^18^F-FDHT). ^18^F-FDHT-PET enables detection of metastatic CRPC with overexpressed AR and indicates a low pharmacological efficacy of ADT (101). Another commonly applied strategy is represented by the utilization of multiple radiolabeled choline derivatives such as ^11^C-methyl-choline and ^18^F-fluorocholine (102). Choline is phosphorylated by the choline kinase overexpressed in PCa and necessary for malignant transformation (103). ^11^C- and ^18^F-choline-PET demonstrated clinical benefit for the detection of bone and lymph node metastases. However, in the latter case, the sensitivity is strongly dependent on PSA level as demonstrated by detection rates of less than 50% for PCa patients with serum PSA level <2 ng/ml (104). Moreover, anti-1-amino-3-^18^F-fluorocyclobutane-1-carboxylic acid (^18^F-FACBC, Axumin^®^, Blue Earth Diagnostics) was proven to be superior to ^11^C-methyl-choline in PET imaging for PCa patients with biochemical relapse after radical prostatectomy (105). Finally, ^18^F-sodium fluoride (Na^18^F) is a hydroxyapatite-affine bone-seeker which is incorporated at sites of active bone remodeling adjacent to metastatic foci analogically to 99m-technetium medronic acid (99mTc-MDP) used for skeletal scintigraphy (106). However, ^18^F-NaF-PET was shown to have a higher sensitivity and specificity for the detection of osseous metastatic disease compared to scintigraphy (107).

### Radiopharmaceutical Options

Among all previously mentioned radiotracers for PCa imaging, particular attention is given to radiotracers targeting the peptidase prostate-specific membrane antigen (PSMA) (108). PSMA expression reflects the progression of the disease, with the highest expression level in the late stage of metastatic CRPC, and enables monitoring of disease recurrence (109). Diverse PSMA-directed antibodies, antibody-derivatives, peptides, peptidomimetics, small molecules, and nanoparticles have been designed as capable diagnostic, therapeutic, and/or theranostic constructs for the management of PCa (110–113). As reported by Zippel et al., more than 100 clinical trials utilize PSMA-specific diagnostics or therapeutics currently (114). Until now, it has been shown that ^68^Ga-PSMA-PET outperforms all standard-of-care imaging within sensitivity and specificity for PCa detection (115). In the randomized proPSMA trial for primary staging of localized high risk prostate cancer, PSMA-based PET imaging showed superior sensitivity and specificity over conventional imaging for accurate diagnosis of nodal and distant metastases {27% (95% CI 23–31) vs. 65% [60–69]; p<0·0001}. Further, ^18^F-PSMA-PET has a significant impact on PCa patient management as shown by a prospective clinical study (116). The most prominent diagnostic radioligand for the imaging of PSMA-positive PCa is ^68^Ga-PSMA-11 (117). Comprehensive meta-analysis by Perera et al. demonstrated high PCa detection rates for ^68^Ga-PSMA-PET with 59% for patients with low PSA levels of 0.5–0.99 ng/ml, 75% for 1–1.99 ng/ml and 95% for PSA values >2 ng/ml (118). In parallel, ^18^F-labeled PSMA ligands like ^18^F-DCFPyL (119) and ^18^F-PSMA-1007 (120) may gain even more clinical importance. For example, ^18^F-PSMA-PET/CT was able to visualize metastatic lesions in >70% of CRPC patients that were not previously detected (121) and in >67% patients with biochemical recurrence whose conventional imaging has also failed (122). On the other hand, 5%–10% of patients with primary PCa are PSMA-negative and PSMA-targeted diagnosis is not applicable in those patients (123). Additionally, patients who receive long-term ADT demonstrate a significant reduction in PSMA expression (97). In this scenario, other targets such as gastrin-releasing peptide receptor (124), fibroblast activation protein (125), and somatostatin receptor (126) demonstrated clinical potential (**Table 1**).

**Table 1:** Clinical trials applying radiopharmaceutical in PCa patients, including patient characteristics, therapeutics, outcome, study ID.

Compound	Characteristics & number of participants	Patient characteristics	Primary outcome measures	Completion date	Study ID & short name
[^68^Ga]Ga-PSMA-11 *compared to histopathology*	Diagnostic Phase I/II 173	Patients with newly diagnosed PCa and a high risk for metastasis, scheduled for radical prostatectomy (RP) with extended pelvic lymph node dissection (EPLND).	True positive fraction (TPF) and false positive fraction (FPF) of identified tumor tissue in soft tissue, analyzed separately for prostate gland and pelvic lymph nodes, using histopathology as standard of truth. Frequency of occurrence and severity of abnormal findings in safety investigations.	Jul 2020	NCT03362359
[^68^Ga]Ga-PSMA-11 *compared with pathology reports and/or routine imaging*	Diagnostic Phase n.d. 1574	Subjects with high risk PCa at initial presentation, with biochemical persistence of PCa following radical prostatectomy, with biochemical recurrence of PCa following initial curative treatment with radical prostatectomy or radiation therapy, with biochemical recurrence of PCa following radical prostatectomy	Sensitivity of [^68^Ga]Ga-PSMA-11 PET/CT imaging in the assessment of high risk and recurrent PCa. Determination of sensitivity when compared with pathology reports (if available) and routine imaging (CT, MRI, bone scan) if available.	Sep 2028	NCT04484701
[^18^F]DCFPyL *compared to histopathology*	Diagnostic Phase II/III 385	Patients with at least high risk PCa who are planned for radical prostatectomy with lymphadenectomy (Cohort A) or patients with locally recurrent or metastatic disease willing to undergo biopsy (Cohort B).	Sensitivity and specificity of [^18^F]DCFPyL PET/CT imaging to detect metastatic PCa within the pelvic lymph nodes relative to histopathology.	Jul 2018	NCT02981368 “OSPREY”
[^18^F]DCFPyL *followed by biopsy/surgery, conventional imaging or locoregional RT*	Diagnostic Phase III 208	Patients with suspected recurrence of PCa who have negative or equivocal findings on conventional imaging.	Correct localization rate, defined as % of subjects with a one-to-one correspondence between localization of at least one lesion identified on [^18^F]DCFPyL PET/CT imaging and the composite truth standard.	Aug 2019	NCT03739684 “CONDOR”
[^18^F]PSMA-1007 *vs. [^18^F]fluorocholine*	Diagnostic Phase III 200	Patients with suspected biochemical recurrence of PCa after previous definitive treatment for localized PCa.	Comparison of detection rate of metastatic PCa lesions for [^18^F]PSMA-1007 versus [^18^F]fluorocholine.	Sep 2020	NCT04102553
[^177^Lu]Lu-PSMA-617 *vs. cabazitaxel*	Therapy Phase II 201	Patients with mCRPC who have progressed despite hormonal therapy and chemotherapy.	PSA RR defined as the proportion of participants in each group with a PSA reduction of ≥50% from baseline.	Jan 2021	NCT03392428 “TheraP”
[^177^Lu]Lu-PSMA-617 *vs. best supportive/standard care*	Therapy Phase III 750	Patients with progressive PSMA-positive mCRPC who received at least one novel androgen axis drug and were previously treated with one to two taxane regimens.	OS in patients with progressive PSMA-positive mCRPC who receive [^177^Lu]Lu-PSMA-617 in addition to best supportive and/or standard of care.	Sep 2021	NCT03511664 “VISION”
[^177^Lu]Lu-PSMA I&T *vs. standard care*	Therapy Phase II 58	Patients with hormone-sensitive oligo-metastatic PCa.	To compare the fraction of patients that have disease progression and meet EOT 1 criteria in a group of patients that are treated with [^177^Lu]Lu-PSMA I&T and a control group.	Jan 2024	NCT04443062 “Bullseye”
[^225^Ac]Ac-PSMA-617 *pilot trial for therapy*	Therapy Early phase I 20	Patients with mCRPC who were incapable of 2^nd^ ADT or chemotherapy.	Serum PSA level.	Dec 2021	NCT04225910
[^225^Ac]Ac-J591 *dose escalation*	Therapy Phase I 42	Patients with documented progressive mCRPC.	Change in the number of subjects with dose limiting toxicities. Estimation of maximum tolerated dose.	Jul 2024	NCT03276572
[^225^Ac]Ac-J591 *dose escalation*	Therapy Phase I/II 105	Patients with progressive mCRPC.	Change in the number of subjects with dose limiting toxicities. Estimation of cumulative maximum tolerated dose. Assessing the recommended phase II dose (RP2D) of [^225^Ac]Ac-J591 in fractionated dose and multiple dose regimens (phase I).	Jun 2027	NCT04506567
[^131^I]-MIP-1095 *with or without enzalutamide*	Therapy Phase II 175	Patients PSMA-avid mCRPC who have progressed on abiraterone and are planned for treatment with enzalutamide. Patients must be chemotherapy-naive and must be ineligible or refuse to receive taxane-based chemotherapy at time of study entry.	The proportion of patients with PSA response according to PCWG3 criteria defined as the first occurrence of a 50% or more decline in PSA from baseline, confirmed by a second measurement at least 3 weeks later.	Dec 2022	NCT03939689 “ARROW”

### Imaging and Theranostic of Skeletal Metastasis

The skeletal compartment is the most frequent site of metastases in PCa patients (127). Bone metastases occupy a nutrient-rich niche that enhances the treatment-resistance of disseminated PCa (128). Approved agents for palliative therapy of PCa patients with bone metastasis include beta-emitting particles such as strontium chloride (^89^Sr-chloride) (129) and samarium-153-ethylene-diamine-tetra-methylene-phosphonate (^153^Sm-EDTMP) (130). However, both options did not improve overall survival and demonstrated limited tolerability due to side effects on the bone marrow and hematopoietic system. On the other hand, alpha-emitting particles including agents such as radium-223 dichloride (^223^RaCl2, Xofigo^®^, Bayer Healthcare) revealed overall survival benefit and reduced symptomatic skeletal events (131). The ALSYMPCA trial reported that the application of ^223^RaCl2 increases median overall survival from 11.3 to 14.9 months and time to develop skeletal-related events from 9.8 to 15.6 months (132).

The novel concept of theranostic approaches combines diagnostics with therapy. Due to the increased availability of potent PSMA-directed agents, several PSMA-labelled radiopharmaceuticals are used in the late stage of PCa. Meanwhile, beta-particle-emitting ^177^Lu-PSMA-617 (133–136) and alpha-particle-emitting ^225^Ac-PSMA-617 (137–139) became the main candidates for PSMA-targeted radioligand therapy of patients with metastatic CRPC. A retrospective multicenter phase I study with 145 patients demonstrated safety and efficacy of ^177^Lu-PSMA-617. The clinical benefit exceeded those of other third-line systemic therapies and prolonged the overall survival in patients without any other treatment option (134, 140–142). A prospective single center phase II trial validated the high response rate, low toxicity, and improved quality-of-life in additional 50 patients for the ^177^Lu-PSMA-617-based theranostic (143). The long-term follow-up of this study including re-treatment upon progression demonstrated higher response rates than other third-line therapies, as far as such comparison between different studies is valid (144). A systematic review from von Eyben et al. concluded that ^177^Lu-PSMA-targeted radioligand therapy decreased PSA level in patients twice as often as chemotherapy (145). Another agent, the ^225^Ac-PSMA-617, revealed an even higher radiological and biochemical response rate in patients with poor prognosis. However, those patients experienced an increased rate of severe side-effects like irreversible xerostomia (139). The current focus is given to the prospective international multicenter phase-III trial called VISION (NCT03511664) which evaluates ^177^Lu-PSMA-617 for the treatment of 750 patients with progressive PSMA-positive metastatic CRPC (146). The outcome of this clinical trial might clarify the role and clinical potential of ^177^Lu-PSMA-targeted radioligand therapy for the management of metastatic CRPC as second-line therapy in the future.

## Radiotherapy for Patients With Metastatic Prostate Cancer

### Clinical Potential of Radiotherapy for Metastatic Prostate Cancer Patients

The current standard-of-care for patients with metastatic PCa includes systemic androgen-deprivation therapy with or without docetaxel-based chemotherapy. The effects of local radiotherapy for men with metastatic PCa as well as the optimal combination with systemic therapies are currently under debate. In particular, the heterogeneity within PCa patients in terms of tumor volume, metastatic distribution, tumor properties, and clinical symptoms impact tumor progression and therapeutic outcome and need to be further investigated. Several ongoing prospective randomized trials aim to clarify the impact of local radiotherapy in patients with metastatic PCa (NCT01957436, NCT03678025, NCT01751438). The randomized phase 3 trial STAMPEDE compared standard-of-care with external-beam radiotherapy to the prostate in metastatic patients and showed no improved overall survival in the whole cohort (HR 0.92, 0.80–1.06; p=0.266). However, in a pre-specified subgroup analysis of patients with low metastatic burden, the trial demonstrated an improved 3-year overall survival in patients with low metastatic burden (819 of 2061 randomized patients) compared with standard-of- care (81% vs. 73%; HR 0.68, 95% CI 0.52–0.90; p=0.007) (147). Within this study, high-volume metastatic disease was defined as presence of visceral metastases and/or more than four bone metastases with at least one outside of the vertebral column and pelvis. These results are in line with the data obtained within the HORRAD trial, the only published randomized-controlled trial so far that has found a survival benefit in men with low metastatic burden applying local radiotherapy in combination with androgen-deprivation therapy for PCa patients with primary bone metastasis (148). This indicates that patients with few metastases could potentially benefit from local prostate radiotherapy. In both trials, only conventional staging such as bone scan or CT was used. As modern PSMA-PET would be able to detect even smaller metastatic lesions, the method has the potential to precisely define low-volume disease. Furthermore, more patients would be staged as high-volume disease. Therefore, the definition of high-volume disease and the question which of those patients would benefit from local radiotherapy has to be addressed in randomized controlled trials in the future. However, there is an urgent need to clarify the benefit of local radiotherapy on metastatic spread not only from the clinical point of view but also from a better understanding of the underlying molecular and cellular mechanisms.

### Clinical Features of Oligometastatic Prostate Cancer Patients

The term oligometastatic cancer refers to a wide range of patients with a low number of metastatic lesions. The occurrence of one to five metastases in those patients leads to a distinct clinical prognosis compared to patients with widespread metastatic disease (149, 150). Oligometastatic patients benefit from local ablative treatment to all visible lesions in terms of a significant clinical benefit for overall survival, time to initiation of systemic therapy, or time to progression (151–156). In general, the prognosis of patients differs when addressing the timepoint of metastatic onset e.g. in patients with oligo-recurrence after initial local therapy, appearance of metastases after local therapy without a local recurrence, or detection of additional metastatic lesions in patients with metastatic disease. It is hypothesized that those differences may be due to primary location and histology, previous treatments, metastasis activity (synchronous metastases vs. metachronous metastases), and metastasis status (lymph node vs. other sites) at first diagnosis (157). Until now, no clinical data are available evaluating the prognostic differences in PCa patients with oligometastatic disease, underlining an urgent clinical need for the development of biomarkers to stratify this heterogeneous group of oligometastatic PCa patients. Another assumption currently under discussion is whether treating all metastatic lesions with ablative intent using e.g. high dose radiotherapy, surgery, thermal ablation or laser resection may lead to complete tumor response, high cure rates, or long-term disease control in a subgroup of oligometastatic PCa patients. This is supported by clinical trials showing a significant benefit in prolonging time to initiation of androgen-deprivation therapy (13 vs. 21 months) or tumor progression after metastasis-directed therapy (MDT) in comparison to standard of care (158).

Due to the development of novel imaging techniques for PCa patients, as already introduced previously, the detection of metastases is possible even at low PSA serum levels (1–2 ng/ml) (159). PSMA-PET-based staging entered successfully the clinical routine for primary diagnosis in high-risk PCa patients and influenced significantly the choice of treatment (160). Moreover, it is applied for staging of patients with biochemical recurrence after prostatectomy or progression after radiotherapy (161). Detected metastases are typically small and asymptomatic in the lymph node or bone. High precision conformal radiotherapy techniques such as stereotactic body radiotherapy is able to control those lesions without significant normal tissue toxicity (162).

PCa with recurrent disease is usually not accompanied by fast progression into symptomatic stages. Patients with recurrence develop symptomatic metastases within a median time of 8 years and a mean overall survival rate of 5 years upon onset. Only a small subgroup of patients characterized with an initial Gleason score of 8 to 10, biochemical recurrence within 2 years, and a PSA doubling time <10 months show a faster metastatic progression (163). In summary, the prognosis of oligometastatic PCa is heterogeneous as those lesions appear at different disease stages at primary diagnosis and upon different pre-treatment regimens. Stratifying those heterogeneous patient population into several subgroups solely based on PSA level is currently under investigation. Unfortunately, no prognostic biomarker for those patients is available so far. Moreover, the development of predictive biomarkers for metastasis-directed therapy would help to answer the clinical questions, if PCa patients would benefit in all stages of the disease (164).

### Metastasis-Directed Radiotherapy

In incurable disease stages, palliative radiotherapy in few fractions is frequently applied to alleviated symptoms including pain, bleeding, or urinary tract problems. The gained improvement of these clinical symptoms, however, does not affect overall survival and metastatic progression at other sites (165). Novel imaging techniques enable the detection of single or few PCa metastases even in patients with low PSA-level and the treatment of those lesions with local ablative radiotherapy (162). Therefore, a growing number of patients are treated with the so-called metastases-directed therapy, including all forms of local treatments (e.g., lymph node dissection, thermal ablation, surgery, or high-dose radiotherapy) with the aim of long-term tumor control. Improved radiotherapy planning systems and precise delivery techniques allow metastasis-directed, local ablative radiotherapy with a few high-conformal fractions as stereotactic body radiation therapy (SBRT). Due to the non-invasive nature of SBRT, the treatment can be done without serious side effects. Most retrospective case series [summary in (150)] focus on a local control and disease progression and demonstrated clinical benefit with local control rates of >90% within the first year. However, further biochemical or metastatic progression after 1 year is observed in ~50% of the treated patients. All published data are not comparable, because those cohorts differ within risk group stratification, primary treatment, concurrent medication, diagnostics, and fractionation scheme. To date, only two randomized trials, the STOMP, and ORIOLE study investigated the clinical benefit of metastasis-directed radiotherapy in comparison to observation as standard-of-care in castration-sensitive PCa patients. Within the STOMP study, 5 out of 31 patients received pelvic lymph node resection and showed a significant improvement of androgen deprivation therapy-free survival (21 vs. 13 months). Within the ORIOLE study, SBRT was applied with a fractionation schedule depending on the metastatic site and included 3 to 5 fractions with a total dose of 19.5–48 Gy. The primary clinical endpoint was progression at 6 months from randomization and proofed safety and efficacy of SBRT to all metastases. The results demonstrated in 19% vs. 61% of the patients a metastatic progression favoring the SBRT arm. However, in both trials, a high number of patients showed biochemical or metastatic progression within 2 years upon locally applied metastasis-directed therapy (166, 167). Due to the rapid progression in the majority of the analyzed patients, the impact of other clinically relevant endpoints, e.g., overall survival, time to castration-resistance, or time to symptomatic progression, remains unclear (168, 169) and should be evaluated in future trials. Moreover, there are still several open clinical questions regarding the treatment of patients with hormone-sensitive, metastatic PCa:

1. What is the optimal radiotherapy volume, as retrospective data indicate fewer nodal recurrences with larger pelvic irradiation fields compared to small node fields (170)?
2. What is the clinical effect and duration of concurrent androgen-deprivation therapy since retrospective data demonstrate a benefit in terms of time to biochemical progression (171)?
3. Can “omics” (e.g., based on tissue or imaging) or other biomarkers guide individualized treatment decisions?

Up to now, the clinical utility of metastasis-directed radiotherapy in patients with oligometastatic CRPC was only demonstrated in retrospective studies. These promising results illustrate that PSMA-based imaging can identify oligometastatic disease in up to 75% of patients when applied at low PSA values (172). Moreover, it was shown that local radiotherapy is able to control or induce regression of the detected metastatic lesions (173–176). The clinical aims of metastasis-directed radiotherapy in terms of long-term curation, regression, or time prolongation of symptomatic disease are currently a matter of debate. However, prospective and randomized clinical data are necessary to demonstrate the clinical benefit of metastasis-directed radiotherapy including clinical endpoints such as velocity of progression, progression of asymptomatic to symptomatic metastases, and overall survival. Nonetheless, the sensitivity and clinical applicability of novel imaging modalities are limited and combination with molecular diagnostics would be necessary in the future for therapy monitoring and early detection of metastatic spread.

## Circulating Tumor Cells in Prostate Cancer

### Biology of Circulating Tumor Cells

**Figure 3:**
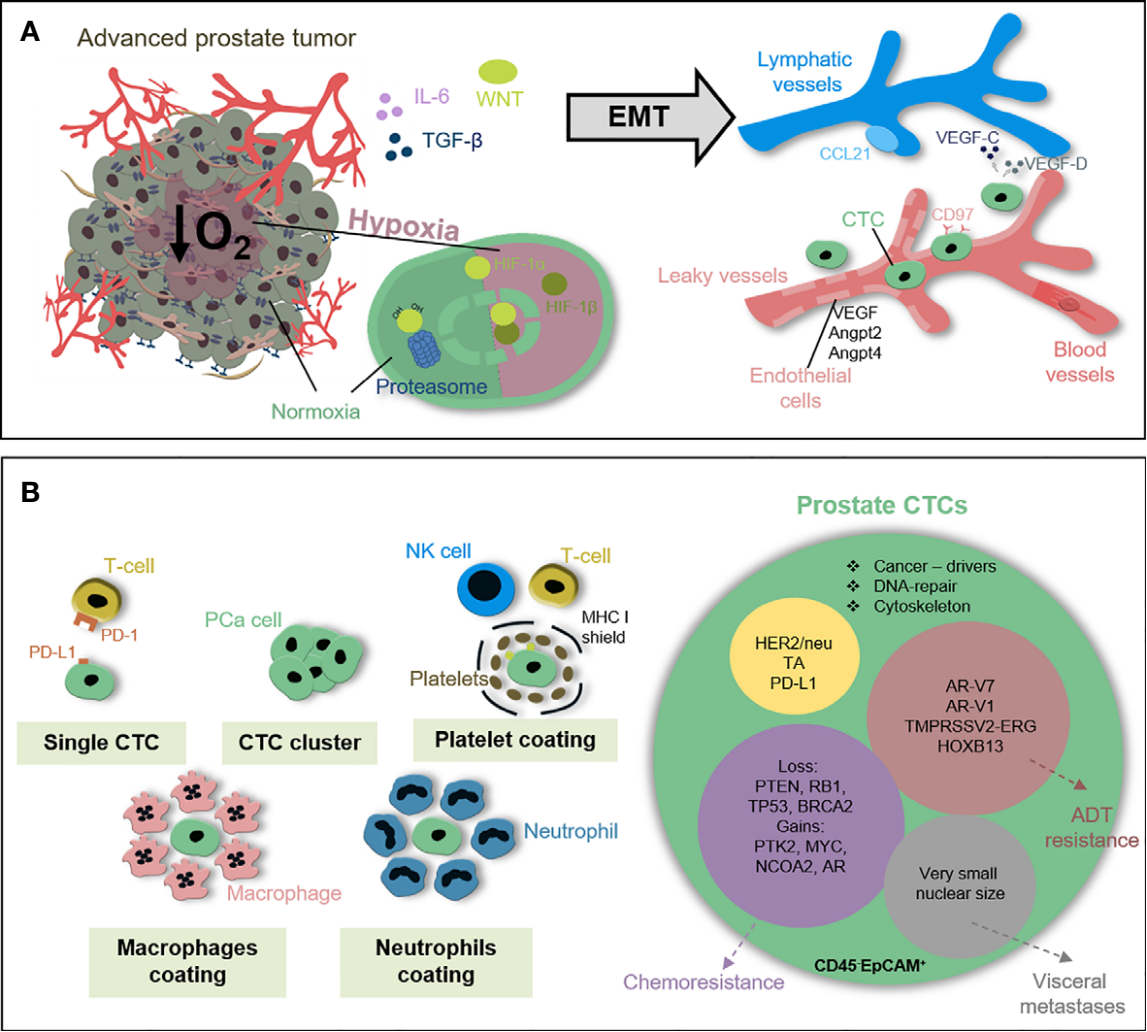
Circulating tumor cells in prostate cancer patients. **(A)** Early metastatic features within PCa cells can be induced under stress conditions e.g. hypoxia, immune attack, or therapeutic pressure. In response to TGF-β, Wnt or IL-6 PCa cells undergo EMT to gain motility and invasiveness. PCa cells intravasate into blood vessels either passively throughout leaky vessel walls or actively *via* trans-endothelial migration. **(B)** Prostate CTCs circulate either as single cells, CTC cluster, or coated with platelets, neutrophils or macrophages shielding immune attack and reducing shear stress. CD45^-^EpCAM^+^ CTCs are a heterogeneous population differing in, e.g. the expression of androgen receptor splice variants, TMPRSS2-ERG status or loss of tumor suppressors PTEN, RB1, and TP53 recapitulating local tumor heterogeneity, influencing metastatic capacity and indicating therapy response.

Circulating tumor cells (CTCs) are malignant epithelial cells within the blood of cancer patients and origin either from the primary tumor or from distant metastasis (177, 178). They were first described in 1869 by the Australian physician Thomas Ashworth (179). The initiation of tumor cell dissemination from the primary tumor is promoted either actively or passively due to tumor cell shedding into surrounding blood vessels during biopsy, surgery, or brachytherapy. Active dissemination is induced through TGF-β, Wnt, or IL-6 stimulation leading to induction of a partial EMT phenotype (180, 181). Upon leaving the primary tumor, migratory cancer cells can intravasate into the blood stream passively through disorganized and leaky vessels in fast growing tumors, which are formed rapidly upon VEGF-induced neovascularization (182–184). In addition, trans-endothelial migration along a chemoattractant gradient consisting of VEGF-C, VEGF-D, and CCL21 regulates active intravasation. In addition, upon adhesion of cancer cells to endothelial cells they secrete cytokines and growth factors, such as VEGF, angiopoietin 2 (Angpt2), and angiopoietin-like 4 (Angptl4), leading to hyperpermeability of the endothelial wall (185). In prostate CTCs, the G-protein coupled receptor CD97 was identified as key promotor for trans-endothelial migration through platelet activation, ATP release, and lysophosphatidic acid signaling (186). Moreover, these platelet coating shields the major histocompatibility complex class I (MHC I) signal and protects CTCs from T and NK cell-mediated immunity. Other groups could demonstrate that CTCs express programmed death-ligand 1 (PD-L1), a member of the B7/CD28 co-stimulatory receptor family, that mediate immune tolerance upon binding to PD-1 on T cells (187). Even nuclear PD-L1 expression in prostate CTCs was found to be associated with poor overall survival of patients (7). Within the circulation, CTCs travel either alone, as cluster, or covered with platelets, megakaryocytes, or neutrophils. In breast cancer, it was shown that CTCs form clusters through the cell junction component plakoglobin or the glycoprotein CD44. Such oligoclonal CTC clusters are better protected from reactive oxygen species (ROS) and exhibit a significantly increased metastatic potential (188). Most of the CTCs entering the circulation die within 24 h either *via* anoikis or immune attack (8). The mean CTC frequency is assumed to be approximately 1 CTC per 1 billion red blood cells with a determined half-life of 2.5 h for breast CTCs (189). The ExPeCT (Exercise, Prostate Cancer, and Circulating Tumor Cells, NCT02453139) trial analyzed the impact of a structured exercise on metastasis progression in PCa patients including analysis of CTCs, CTC clusters, and platelet-CTC cloaking. So far, there are no study results published, but preliminary analysis demonstrated no relationship between physical exercise and CTC count. However, first indications point to a significant influence of immune crosstalk on metastasis cascade (190). In breast cancer patients, Szczerba et al. analyzed CTC-associated white blood cells and found a connection with neutrophils. CTCs within cluster, together with neutrophils, display differently regulated genes involved in cell cycle progression, cell-cell junction, and cytokine receptor expression, survive better in the blood stream, and exhibit elevated metastatic potential compared to single CTCs (191). Active CTC extravasation is induced by rolling of CTCs along the endothelium mediated by interaction with CD44 and integrin αvβ3 (192). In addition, hemodynamic forces facilitate adhesion of CTCs to the blood vessel wall and induce endothelial remodeling (193). Upon stabilization of CTC-endothelium interaction, CTCs induce extravasation through binding of sialofucosylated proteins, such as podocalyxin or glycosphingolipids with C-type lectin binding, e.g., E-selectin (CD62E), on endothelial cells (194). Besides the above described, TGF-β induced hematogenous dissemination and lymphatic spreading was described for several tumor entities including colorectal cancer (180, 195) (**Figure 3A**). A recently published study demonstrated protective metabolic priming of melanoma cells within the lymph node and increased metastatic potential. The metabolic rewiring is mediated by oleic acid within the lymph node and reduces oxidative stress, lipid oxidation, and ferroptosis when the cancer cells travel through the blood stream (196). It is not known whether this protective metabolic mechanism is also involved during lymphatic spread of PCa.

Within local PCa tumor heterogeneity and cellular plasticity are key regulators for progression, therapy resistance, and metastatic spread (197–199). A population with a high degree of heterogeneity has a higher chance to survive evolutionarily (200, 201). Recent findings indicate that the prostate CTC population is heterogeneous in terms of their genomic alterations, gene expression profile, and cell surface marker expression (202–204). Lack of datasets correlating the impact of CTC heterogeneity and plasticity for metastatic spread and therapy response in PCa patients is a consequence of low CTC number and limited availability of molecular approaches with high sensitivity and specificity (205). This obstacle was tackled by the group of Johann de Bono which isolated prostate CTCs from patients with lethal disease based on apheresis technique. Therapeutic apheresis removes patient’s blood followed by the separation of cells-of-interest from other blood cells, e.g., in the mentioned study of EpCAM^+^ CTCs, and reinfusion of the blood. With the application of this method, the group was able to isolate app. 12,500 CTCs per patient within 59.5 ml blood. 185 single CTCs from 14 patients underwent genomic analysis *via* array-based comparative genomic hybridization upon whole genome amplification. The individual copy number alteration demonstrated complex intra- and interpatient heterogeneity (202). However, all published results analyzing prostate CTCs are not experimentally homogenized according to the isolation procedure and biomarker analysis (206). Therefore, the European cancerID consortium (2014–2019) was aiming to establish clinical utility of liquid biopsy analysis (207, 208). The study by Massard et al. impressively demonstrated how isolation methods affect CTC count and characterization. This group compared two CTC isolation techniques, CellSearch with isolation by size of epithelial tumor cells (ISET) filtration, and found that the CellSearch system is biased to identify CTCs with epithelial phenotype while missing mesenchymal CTCs and CTC cluster. However, detection rate of AR amplification based on downstream fluorescence *in situ* hybridization analysis was higher in CellSearch enriched CTCs compared to ISET (209). Another study published by Scher et al. investigated the heterogeneity of prostate CTCs in 179 patients with metastatic disease and how the degree of CTC heterogeneity can be clinically applied to support decision making either for AR inhibitor-based therapy or taxane-based chemotherapy. They hypothesized that the degree of pre-therapeutic CTC heterogeneity inversely correlates with overall survival upon ADT but not with chemotherapy. Therefore, they analyzed cells within the blood upon red blood cell lysis using automated immunofluorescent analysis for nuclear DAPI, leukocyte marker CD45, epithelial marker cytokeratin (CK), and prostate-specific AR. Upon digital pathology, the Shannon diversity index describes the occurrence of individual CTC clones within the whole CTC population defined as DAPI^+^CD45^-^. Heterogeneity was evaluated based on densitometric, morphometric, and texture patterns of nuclear DAPI, CK, and AR signal. The results validated the relationship between the degree of CTC heterogeneity and overall survival for ADT, but not for taxanes. In addition, genomic profiling of 10 CTCs in 17 patients identified unique driver subclones for ADT resistance (210). Further studies validated the clinical utility of molecular CTC features for clinical decisions. For example, the expression of the AR splice variant 7 (*ARv7*) status in CTCs of metastatic CRPC patients is able to predict the efficacy of ADT (211–213). So far, no published study correlated CTC heterogeneity and dynamics with predictive value for radiotherapy response and metastatic progression in PCa patients.

### Clonal Evolution and Dynamics Within Prostate Circulating Tumor Cells

That tumors follow the Darwin’s theory of evolution was already proposed by Peter Nowell in 1976. This can be seen in slow growing PCa which is characterized by extensive intra-tumoral heterogeneity and sub-clonal diversity (74, 214). This clonal diversity has a significant impact on therapy response. For example, Beltran et al. analyzed 114 biopsies from 81 patients with metastatic CRPC including specimens with adenocarcinoma (Adeno) or neuroendocrine (NE) features. The differentiation into neuroendocrine morphology includes the downregulation of AR and explains the ADT escape. The genome-wide expression and DNA methylation data of this study demonstrated a high level of clonality, but overall similarity of genomic alterations while epigenetic adaptations were able to distinguish CRPC-Adeno from CRPC-NE subset. Key mechanisms important for the induction and maintenance of the ADT-resistant state base on cell-cell adhesion, EMT and histone methyltransferase EZH2 signaling. These findings support the independent emergence of an AR-insensitive cell state through clonal evolution as major ADT resistance mechanism (214). Several studies demonstrated that this clonal heterogeneity and genomic alteration known from stepwise prostate tumorigenesis could be recapitulated within the CTC population including the detection of tumor suppressor gene loss, e.g. *PTEN*, *RB1*, and *TP53* (215, 216) (**Figure 3B**). Moreover, Mahili et al. determined copy-number alteration in 257 isolated CTCs from 47 patients with aggressive PCa treated with cabazitaxel- and carboplatin-based chemotherapy and found a higher frequency of detectable chromosomal alteration in CTCs compared to match-paired cell-free tumor DNA (73.7% vs. 42.1%). The observed genomic instability in CTCs is independent of the CTC count and associated with chromosomal gains in regions containing the *PTK2*, *MYC*, and *NCOA2* gene increased AR expression, and *BRCA2* loss (217). This opens new preclinical and clinical questions:

1. Does molecular analysis of CTCs have the potential to predict sites and degree of metastatic spread?
2. How does the genetic profile of CTCs overlap with metastases and are CTCs the origin of polyclonal metastatic lesions?
3. Do CTC-based analysis outcompete routine diagnostics such as PSA plasma level, Gleason score or imaging modalities to predict and monitor therapy response in PCa patients, in particular for local or systemic metastasis-directed therapies?

To demonstrate the clinical importance of CTCs for the diagnosis of metastasis, Faugeroux et al. performed whole-exome sequencing analysis from 179 isolated CTCs and matched metastasis biopsies from 11 PCa patients. They found that app. 30%–50% of the mutations are shared between the metastasis and epithelial CTCs. In addition, a CTC exclusive mutation pattern was found in epithelial and non-epithelial CTCs containing known cancer-driver genes and genes involved in cytoskeleton and DNA repair. Based on these data, the group hypothesized that the phenotypically distinct CTC populations found in the patient’s blood resemble a phylogenetic relationship rather than offspring from different precursors (218). Another study was able to distinguish three morphologically distinct CTC populations based on nuclear size measurements. Upon analysis of 148 blood samples from 57 PCa patients, they were able to identify patients with visceral metastasis based on the amount of very small nuclear CTCs (219). However, further experimental studies and prospective clinical trials are needed to prove clinical utility of CTC diagnostics and answer upcoming clinical questions e.g. in terms of decision-making for metastasis-directed therapy, in particular for oligometastatic PCa patients with ablative radiotherapy. Cell-extrinsic pressures, such as environmental forces, immune attack, or lack of nutrients are key drivers for clonal evolution and cellular plasticity influencing the degree of tumor heterogeneity. Therapeutic pressure is another driver for clonal selection and induction of cellular escape mechanisms influencing geno- and phenotype of CTCs. Novel findings indicate that different CTC populations may have different metastatic potential in terms of frequency and site-specificity.

### Clinical Application of Circulating Tumor Cell-Based Diagnostics

The detection of ≥5 CTCs per 7.5 ml blood in PCa patients with metastatic disease has a relevant prognostic value and correlates significantly with reduced progression-free survival and overall survival compared to patients with <5 CTCs (220–222). This data led to the approval of CTC-based diagnostics *via* CellSearch system by the United States Food and Drug Administration (FDA) in 2008 and the implementation into recommendations by international trial groups like Prostate Cancer Working Group (PCWG), Southwest Oncology Group (SWOG by National Cancer Institute) and European Organization for Research and Treatment of Cancer (EORTC). Most of the published studies applied the CellSearch system with a phenotypic definition for prostate CTCs as leukocyte marker CD45-negative and epithelial cell adhesion molecule (EpCAM)-positive. Despite the presence of CTCs in PCa patients can be correlated with prognosis and metastatic status, the predictive value is still under debate. Lowes et al. assessed the presence of prostate CTCs at baseline and several time points after radiotherapy (6, 12, and 24 months) (31). They found no correlation between PSA-level and CTC count. However, the presence of extracapsular extension or seminal vesicle invasion combined with CTC-positive status at baseline was predictive for poor response to radiotherapy. Therefore, determining the number of CTCs during radiotherapy may have the potential to stratify patients that need additional systemic therapy from those with high therapeutic efficacy from local radiotherapy alone. Moreover, neither of the standard parameters such as time to biochemical recurrence, PSA doubling time, and pathological features (e.g. Gleason score or margin status) nor available imaging technologies can provide information about the precise location of upcoming recurrences (223). First clinical indications point to the potential of CTCs to predict metastatic spread even upon therapy and the ability to discriminate different sites of metastasis. Besides promising results for CTC-based diagnostics in PCa patients with metastatic disease (222, 224), the prognostic value of CTC count in localized stages is currently not clear due to low detection rates. Most studies analyzing CTCs in locally-advanced PCa patients applied a reduced cut-off value from five to one CTC per 7.5 ml blood or increased analyzed blood volume. However, the data are controversial and the prognostic values of CTC count within this PCa patient group could not be demonstrated yet (225). A recently published study analyzed CTCs in treatment-naïve patients with locally advanced high-risk PCa (NCT01800058, n=66) (226). The authors found that the baseline CTC count was associated with conversion into stage T3 and N1, but not with overall survival. Initially, CTC-negative patients became CTC-positive directly upon androgen-deprivation therapy or radiotherapy followed by a consecutive drop in CTC count within 6–12 months. The authors hypothesize that passive mechanisms due to tumor destruction are responsible for the observed increase in CTC count directly upon therapy. Another, still recruiting, phase III trial (SABR-COMET 10, NCT03721341, n=159) aimed to analyze the clinical benefit of stereotactic ablative radiotherapy for oligometastatic PCa patients (227). Besides the primary endpoint analyzing overall survival, it is planned to evaluate translational endpoints, such as CTC count or immune cell composition (228). All in all, these data demonstrate that CTC count can be applied as prognostic marker in metastatic PCa patients, but it is still controversial whether it is an independent predictor for overall survival. In combination with other prognostic markers such as albumin, alkaline phosphatase, hemoglobin, lactate dehydrogenase (LDH), and PSA the CTC count was able to discriminate PCa patients independently on their treatment (NCT00638690; NCT01193244) (229). These findings were validated in another study that analyzed CTC count in combination with LDH measurements. Based on both parameters PCa patients could be stratified into a low-risk (<5 CTCs, LDH independent), intermediate (≥5 CTCs, LDH ≤ 250U/L), and high-risk group (≥5 CTCs, LDH>250U/L) (230).

While EpCAM-based CTC enumeration methods may miss CTC subpopulation with low EpCAM expression, there are attempts to apply additional markers for CTC detection to increase sensitivity and specificity or apply label-free methods such as microfiltration, density gradient centrifugation or dielectrophoretic techniques (231, 232). Putative prostate CTC markers include e.g. EMT phenotype (NCT02025413), the tyrosine kinase cMET (NCT02080650), the immune checkpoint marker PD-L1 (NCT02456571), telomerase activity (SWOG Trial S042) (233, 234) and the TMPRSS2-ERG translocation (NCT00485303, NCT00474383) (235). The applicable additional marker would enable the monitoring of therapy resistance in real-time and may recapitulate tumor heterogeneity within the blood. Another putative prostate CTC marker is the human epidermal growth factor receptor 2 (HER-2/neu), but detection level was demonstrated to be higher in metastatic patients compared to local disease (236). Promising results were also obtained with the cytological ISET test in combination with prostate-specific marker PSA and prostein (P501S). Within this observational study, 20 men with diagnosed PCa were analyzed with a mean CTC count of 6.5 CTCs per 7.5 ml blood (237). Interestingly, in patients without previously diagnosed PCa ISET-CTC-based screening demonstrated a predictive value of 99% compared to 25% with the standard PSA-based test method within patients receiving PSMA-PET-imaging later on.

Another important clinical question is the predictive potential of CTCs and the possibility to monitor acquired therapy resistance in real-time. As already mentioned above, clinical data for radiotherapy are limited so far. However, the expression of the androgen receptor splice variant 7 (ARv7) in CTCs of patients with metastatic CRPC is able to predict the therapeutic potential of ADT (213, 238, 239). In addition, the predictive value of other AR splice variant transcripts, e.g., *AR-V1*, *AR-V3*, *AR-V7*, and *AR-V9*, was investigated in comparison to the canonical full-length version in CTCs of metastatic CRPC patients under cabazitaxel treatment (n=118) (CABARESC trial). Although all AR variants were similarly co-expressed at baseline and post-treatment, patients carrying *AR-V9*-positive CTCs display decreased CTC counts below the threshold. In turn, *AR-V1*-positive CTCs after cabazitaxel treatment, but not at baseline, was an independent prognostic factor for reduced overall survival (240). The TAXYNERGY trial found an association of *AR-V7*- and *AR-V567*-negativity in metastatic CRPC patients before taxane therapy with PSA response and progression-free survival. Within those analyses, the authors compared the sensitivity of digital droplet PCR (ddPCR) in comparison to quantitative PCR-based method and found an increased detection rate of *AR-V7* variant with ddPCR (19% to 55%) (241). This method was also applied for prostate CTC detection by Miyamoto et al. and demonstrated that CTC-specific *HOXB13* gene expression may identify patients with altered AR-signaling and disease progression under abiraterone therapy (n=27) in patients with localized PCa (n=34) (242). Approximately 50 ongoing clinical studies (20 terminated, 13 with results) worldwide aim to validate the clinical utility of CTC count for PCa patients undergoing radical prostatectomy (16 studies), androgen-deprivation therapy (16 studies) or radiotherapy (28 studies) and implemented CTC-based diagnostics as secondary endpoint (**Table 2**, www.clinicaltrial.gov). In the upcoming years, the results from the running clinical trials may prove the potential of CTC-based diagnostics for patient stratification and therapeutical decision making. Furthermore, CTCs may help to identify patients with a high risk to develop metastasis even at the early stage of the disease and maybe predict the site of metastases occurrence before they are detectable with imaging.

**Table 2:** Summary of completed clinical trials applying enumeration of circulating tumor cells (CTCs) in PCa patients either as primary or secondary endpoint.

Treatment	CTC detection method	Study type & number of participants	Patient characteristics	CTC-specific endpoint	Completion date	Study ID & short name
Cryosurgery with or without dendritic cells and cytokine-induced killers	Flow cytometry, RT-PCR	Observational (n=60)	PCa patients with stages II, III, IV	CTC count within 6 months	Dec 2015	NCT02450435
–	Filtration system	Observational (n=14	Breast cancer, PCa, colorectal cancer patients and healthy volunteers	CTC count	Jan 2014	NCT01943500
ADT, RT	CellSearch	Observational (n=68)	High-risk PCa	CTC count (before treatment, post ADT, 1–3 months post-RT, 6–12 months post-RT)	Dec 2018	NCT01800058
Sipuleucel-T (Provenge), ADT	CellSearch	Observational (n=38)	mCRPC patients with visceral or high-risk disease, metastatic castration sensitive PCa patients with high tumor volume	Expression of immune checkpoint marker PD-L1, PD-L2, B7-H3, and CTLA-4 on CTCs (baseline, 12 weeks, 14 months)	Jun 2019	NCT02456571
–	Ferrofluid EMT-Based Capture Method (CTC-EMT)	Interventional (n=46)	mCRPC, neuroendocrine prostate cancer (NEPC), metastatic breast cancer	CTC detection using mesenchymal-marker N-cadherin or O-cadherin	Dec 2015	NCT02025413
–	Ferrofluid c-MET-Based Capture Method (CTC-MET)	Interventional (n=62)	Progressive metastatic cancer patients	CTC detection using mesenchymal-marker c-MET	Jul 2016	NCT02080650
Docetaxel/Cabazitaxel with prednisone	GEDI ddPCR	Interventional Phase II (n=63)	mCRPC	Reduction of nuclear AR from baseline	Aug 2015	NCT01718353 “TAXYNERGY”
Docetaxel, Prednisone Atrasentan	*Parylene-C slot microfilter, qPCR-TRAP	Observational, Phase III (n=263)	mCRPC	Telomerase expression in CTCs	Jan 2010	SWOG Trial S0421
Abiraterone acetate, prednisone	CellSearch	Interventional Phase III (n=1195)	Docetaxel-refractory mCRPC	CTC count in combination with albumin, LDH PSA, hemoglobin, ALK	Oct 2012	NCT00638690
Orteronel, prednisone	CellSearch	Interventional Phase III (n=1560)	Progressive, therapy-naive mCRPC	CTC count in combination with albumin, LDH PSA, hemoglobin, ALK	Apr 2016	NCT01193244
Cabazitaxel, ADT	Gene expression	Interventional Phase II (n=140)	Docetaxel refractory PCa patients without SCPC or NEPC	CTC count 9–12 weeks after start of treatment	Sep 2019	NCT03050866
Doxorubicin-GnRH agonist conjugate AEZS-108	IF	Interventional Phase I/II 108	PCa patients	AEZS-108 internalization and LHRH expression	Feb 2017	NCT01240629
Cabazitaxel, Prednisone, Ciprofloxacin, G-CSF	unknown	Interventional Phase IV (n=45)	Docetaxel-refractory CRPC grade IV	CTC count (days 42, 84, 126, and post-treatment)	Jan 2014	NCT01649635 “PROSPECTA”
Cabazitaxel, budesonide	CellSearch, RT-PCR	Interventional Phase II (n=118)	mCRPC	Predictive value of AR-V3 and AR-V7 vs. AR-FL expression in CTCs (baseline, post-treatment)	Oct 2015	2011-003346-40 “CABARESC”

ADT, androgen deprivation therapy; ddPCR, digital droplet PCR; GEDI, geometrically enhanced differential immunocapture; G-CSF, granulocyte colony-stimulating factor; IF, immunofluorescence; nd, non-defined; NEPC, neuroendocrine prostate cancer; NSCLC, non- small cell lung cancer; RT, radiotherapy; RT-PCR, reverse transcriptase polymerase chain reaction; SCPC, small cell prostate cancer; TRAP, Telomeric repeat amplification protocol.

## Disseminating Tumor Cells and Minimal Residual Disease in Prostate Cancer

### Early Prostate Cancer Cell Dissemination and Dormancy

Approximately 35% of PCa patients with local disease will develop a recurrence within 10 years and around 10% of those patients present already bone involvement at the time of diagnosis (127, 243–245). This clinical observation indicates that tumor cell dissemination happens at early phases during tumorigenesis without clinical symptoms for decades. However, it is unknown how often and to what extent early dissemination happens upon cellular transformation and tumor initiation. The vast majority of malignant cells leaving the primary tumor are eliminated within the surrounding tissue, the blood stream, or the lymph vessels by immune cells (246, 247). It is hypothesized that <0.01% of metastasis-initiating cells survive in the blood stream with inherent properties to initiate distant metastasis. Therefore, disseminated tumor cells (DTCs) have to switch their phenotype and function from mesenchymal state back to epithelial features, the so-called mesenchymal-to-epithelial transition. In addition, they require a supportive niche including activated stroma and immune suppressive environment (4). The phenotype of prostate DTC is not fully identified yet and might be different within different patient subgroups and upon therapeutic pressure. DTC detection methods apply negative markers to exclude immune cells (e.g., CD45, CD34, CD61) and positive selection for the epithelial cell adhesion molecule (EpCAM). Despite the DTC frequency is low and in most of the analyzed patients below detection level, the prognostic value of prostate DTC is of high clinical relevance to identify patients with increased risk for bone progression and the need for therapeutic adaptation. To address this, Morgen et al. analyzed bone marrow aspirates of 569 PCa patient’s prior radical prostatectomy and compared the DTC count with biochemical recurrence. Therefore, 10 ml bone marrow from the iliac crest was separated using Ficoll-Isopaque-based density gradient centrifugation followed by exclusion of immune cells *via* CD45/CD61-dependent magnetic-associated cell separation and EpCAM-based evaluation with immunofluorescence microscopy. The threshold for DTC positivity was set to ≥1 CD45^-^CD61^-^EpCAM^+^ cell. In 72% of the analyzed patients DTCs were detected already prior to surgery, but without correlation to pathological stage, Gleason score, or PSA level. However, in 98 patients with no evidence of disease after radical prostatectomy, DTC occurrence had a significant predictive value for biochemical recurrence indicating the importance of dynamic diagnostic sampling (248). For independent validation of the clinical findings, it would be critical to develop uniform and standardized prostate DTC detection methods and nomenclature. Besides the established phenotype combining negative markers to exclude hematopoietic lineages and positive marker for epithelial cells, several studies applied also prostate-specific markers to increase specificity and sensitivity. For example, Chalfin et al. analyzed bone marrow aspirates from 208 PCa patients with local disease and compared different DTC detection methods, including antibody-based enrichment with epithelial (e.g., EpCAM) and prostate-specific (e.g. NKX3.1, AR, PSA) markers and found that epithelial markers are not applicable due to unspecific binding (249). A recently published study analyzed the transcriptome of single EpCAM^+^CD45^-^ bone DTCs from prostate cancer patients (77 cells in 10 patients) and distinguished DTCs according to their gene signatures into no evidence of disease (NED) and advanced disease origin. Prostate specificity was validated by prostate-specific markers including AR, CD63, FOLH1, HOXB13, ID1, NKX3-1, RELB, and XAGE1A and the exclusion of erythroid lineage marker. Unsupervised cluster analysis identified p38 stress response pathway regulating dormancy in NED-associated DTCs, which was not found in DTCs of patients with advanced disease. In addition, the authors validated the upregulation of dormancy genes in NED DTCs including ABI1, CDC25B, CDK7, CELF1, and COX7B2 (250). Another study published by Cackowski et al. used fluorescence-activated cell sorting to isolated CD45^-^CD235a^-^AP^-^CD34^-^EpCAM^+^ DTCs and found in 17% of PCa patients (10 out of 58) with local and in 50% with metastatic disease (4 out of 8) >5 DTCs per 10^6^ bone cells. Whole exome sequencing, RNA sequencing, and gene expression analysis identified characteristic single nucleotide polymorphism and gene variants for PCa, but found also a B-lineage-like signature in prostate DTCs indicative of niche adaptations (251). Several previously published studies demonstrated already that prostate DTCs hijack the hematopoietic stem cell niche within the bone marrow to survive quiescence over decades (252). This was elegantly shown by the group of Russel Taichman using an experimental model based on subcutaneous transplantation of human PCa cell lines PC3 and C4-2B in CD45.1-expressing immunocompromised NOD/SCID mice. Upon surgical removal of the subcutaneous xenograft tumor, transplantation of bone marrow cells origin from CD45.2 mice was performed. The authors found that hematopoietic stem cell engraftment was decreased in tumor-bearing mice compared to control and that PCa cells occupy the endosteal niche close to Runx2-expressing osteoblasts (253). Once within the niche, tumor cell dormancy is dictated by the environmental niche factors as well as by tumor cell intrinsic features. For example, Yu-Lee et al. demonstrated cellular quiescence of bone-tropic PCa cell line C4-2B upon culture with conditioned media originated from differentiated and undifferentiated osteoblast cultures. Moreover, Axelrod et al. validated in AXL-null and overexpressing prostate cancer cell lines dormancy induction *in vivo* (254, 255). However, they did not find AXL expression in primary or metastatic prostate tissue and it is questionable if AXL is expressed in DTCs. Beside this described cell-extrinsic cues, cell‐intrinsic features may impact the dormant state of PCa cells. Within a recently published study, Owen et al. demonstrate that type I interferon (IFN) signaling regulates PCa dormancy and metastatic outgrowth in the bone. Therefore, they injected intracardially murine PCa cell line RM1 labeled with the red‐fluorescent dye PKH26 into C57BL/6 mice and isolated red-labeled cells from the bones using fluorescence activated cell sorting. They found that cell intrinsic expression of type I IFN was dynamically regulated on the epigenetic level *via* a histone deacetylase-dependent mechanism. Moreover, they speculate that the observed loss of IFN signaling within the tumor and the suppressed tumor immunogenicity in bone metastases may be an explanation of why current immunotherapeutic strategies fail in patients with metastatic PCa (256). However, certain studies postulate that bone niche and dormancy signaling may be putative therapeutic targets to prevent bone metastasis in PCa patients. These agents include bone homeostasis targeting compounds affecting osteoclast-osteoblast equilibrium e.g., bisphosphonates, the anti-RANKL antibody denosumab, or radiopharmaceuticals such as radium-223. Inhibition of signals within the microenvironment, e.g. *via* ET1 receptor inhibitor, SCR inhibitor (e.g. dasatinib), thalidomide, cabozantinib, or androgen-directed agents demonstrated already clinical benefit in patients with metastatic PCa. However, androgen-deprivation therapy is often associated with bone loss and has a negative impact on the incidence of bone metastases (257). Another possibility to turn dormant DTCs sensitive to chemotherapeutics and to reduce late recurrences would be the re-activation and induction of proliferation. Several studies investigated the underlying molecular mechanisms as putative therapeutic targets. For example, Decker et al. found that the sympathetic nervous system and the neurotransmitter norepinephrine stimulated PCa cell proliferation in the bone niche *via* β2-adrenergic receptors and decreased the secretion of growth arrest specific-6 (Gas6) by osteoblasts (258). However, this strategy is critically discussed due to the risk of further metastasis initiation. Another newly discovered process that might foster tumor growth and metastasis is the so-called tumor self-seeding, a phenomenon where CTCs or re-activated DTCs return to the site of tumor of origin (259, 260). For example, it has been shown that self-seeding CTCs in human osteosarcoma was mediated by interleukin 8-CXCR1/2 axis, resulting in an increased metastatic potential (261). In metastatic PCa, translational and retrospective studies indicate that local treatment to the primary tumor affects metastatic spread and patient outcome. However, the data are controversial, and supportive prospective trials are needed before the implementation of this concept into clinical routine recommendations (262). Data from the STAMPEDE trial shows that radiotherapy to the primary tumors in M1 disease stage improves overall survival of low burden PCa patients by 8% after 3 years [hazard ratio: 0.68, p-value 0.007 (arm H)] (263). However, biomarker research is urgently needed to discriminate metastatic PCa patients profiting from those local therapies. In parallel, experimental and translational studies are necessary to improve our understanding of the underlying molecular and cellular mechanisms regulating early dissemination, metastatic spread, and colonization.

### Liquid Biopsy-Based Methods for Detection of Minimal Residual Disease

Besides early dissemination, another clinical obstacle is the monitoring and treatment of PCa patients with minimal residual disease (MRD). This concept describes remaining tumor cells after initial therapy and complete remission. These few malignant cells and/or micro-metastasis cannot be detected by routine diagnostics, e.g. plasma PSA level or PET imaging. It is hypothesized that they persist locally as cancer stem cells (CSC), in the circulation as CTCs, or at distant organs such as the bone marrow as DTCs. The National Cancer Institute defines MDR as one cancer cell among one million normal tissue cells. First evidence for MDR in PCa was published by Murray et al. as prospective data analysis of 321 patients 10 years after initial radical prostatectomy including CTC and DTC count 1 month after therapy. Based on CTC and DTC positivity, the patients could be stratified into 4 subgroups with significant differences in overall survival. The authors found that CTC positivity correlates with early relapse while DTC positivity is associated with late failure. Therefore, they propose the existence of two forms of MRD representing different clinical characteristics (264, 265). This leads to the hypothesis that the dynamics of MRD determines therapy response and patient outcome. MRD can be analyzed through detection of tumor-specific antigens, genetic and epigenetic changes in bone marrow aspirates and/or peripheral blood with highly sensitive multiparameter flow cytometry, digital droplet PCR, or next generation sequencing (NGS)-based methods. Despite the sensitivity and specificity of molecular genetic methods to detect prostate specific gene fusions, transcript variants, or point mutations in cell-free tumor DNA (cfDNA) is higher (1 cell in 10^6^ cells) compared to antibody-based detection methods determining DTC/CTC count (1 cell in 10^4^ cells), it is cost-intensive and therefore only available for a small subset of patients. Moreover, the mutational load in PCa is compared to other tumor entities relatively low with a somatic mutation rate between 1x10^-6^ and 2x10^-6^. For example, in primary PCa app. 50% of the patients harbor a *TMPRSS2-ERG* gene fusion (70, 266, 267). In metastatic CRPC the mutational burden is app. 3.8-fold higher compared to the earlier disease stages including an increased frequency of driver mutations such as *AR* (5%–30%), *TP53* (3%–47%), and/or *PTEN* (20%–60%) (268). Wyatt et al. compared the mutational pattern of cfDNA with the primary tumor in 45 patients with metastatic PCa and found 88.9% concordance. 75% of the tested patients showed a fraction of circulating tumor DNA (ctDNA) >2% of the total cfDNA. In 64.7% of those patients an AR amplification and in 8.8% a *SPOP* mutations were detected (269). Based on these findings, the authors propose that cfDNA assays are sufficient to identify all driver mutations and may guide clinical decision making for metastatic CRPC in the future. Currently, there is no approved clinically test for prostate MRD available. However, the prognostic potential of those assays is demonstrated by the FDA approval of the NGS-based method cloneSEQ to detect MDR in multiple myeloma, B-cell acute lymphoblastic leukemia and chronic lymphocytic leukemia in 2018. Within the same year, the FDA approved the Oncotype DX AR-V7 Nucleus Detect^®^ test for the detection of the splice variant of the androgen receptor *AR-V7* in CTCs for late-stage mCRPC to predict responsiveness to androgen deprivation. On the other hand, the immunophenotype-based detection methods for CTCs and DTCs still need clinical standardization before they may become broadly available. The disadvantage of this method is the dependency on the detection of pre-defined markers e.g., epithelial markers such as EpCAM which are dynamically regulated during tumorigenesis, clonal evolution, metastatic spread and under therapeutic pressure. Therefore, highly sensitive, label-free approaches based on microfluidic devices to discriminate different cell populations based on cell size or cell viscosity are currently under development and in clinical testing, e.g., the Parsortix^®^ system (ANGLE plc.), the DEPArray™ System (Menarini Silicon Biosystems), the ClearCell^®^ FX System or real-time deformability cytometry (270–273). Additionally, non-invasive tests to monitor tumor progression and therapy response in urinary samples of PCa patients, for example, gene expression analysis of urine exosome with the ExoDx (IntelliScore) test (274). If these approaches can be applied for DTC analysis in the bone has to be tested. Moreover, sensitivity, and specificity, as well as clinical applicability, are necessary before proposing MRD positivity to guide treatment planning and individual decision making for metastatic PCa patients. Moreover, at present, there is no experimental or clinical study published investigating DTC counts and MRD upon radiotherapy. Future prospective clinical trials for MRD detection methods may consider novel clinical endpoints such as metastasis-free survival for non-metastatic CRPC (275). However, given the high degree of heterogeneity within PCa and the dormant cell state of DTCs the applicability of MRD diagnostics in PCa might be limited.

### Impact of the Immune System on Metastatic Spread

Metastasis-initiating PCa cells use the homing factor CXCL12, which is under physiological conditions a chemoattractant secreted by stromal cells and involved in the regulation of bone marrow homing, retention, and mobilization of hematopoietic stem cells (HSC) (253, 276). Despite PCa cells hijack the HSC homing route and bone niche, upon arrival they often enter a dormancy state induced by GAS6 or DKK1 signaling and thus evade immune attack (277). The connection of cancer progression and chronic inflammation was already described in 1863 by Rudolf Virchow who recognized an increased leukocyte count in tumors (278). Today we distinguish ‘hot’ tumors with an inflammatory hallmark based on a high number of infiltrating T cells such as melanoma or lung cancer from ‘cold’ entities. These tumors are genetically unstable, with high mutational burden and increased production of T cell recognized neoantigens. However, PCa is classified as ‘cold’ tumor with a low rate of immune infiltration. At the primary site, tumor cells generate an immune suppressive environment through recruitment of myeloid cells and macrophages to escape from CD8^+^ T cell- and NK cell-mediated cell killing (279). In particular, tumor-associated macrophages (TAMs) are able to switch their phenotype from tumor-suppressive (M1) to tumor promoting (M2) function. M2 TAMs promote migration and environmental adaptations at the metastatic site (280). The interaction of CD163^+^ M2 macrophages and FoxP3^+^CD4^+^ regulatory T cell (Treg) was investigated by Erlandsson et al. in PCa biopsies from 1367 patients with localized tumors. Within this study, they separated patients with tumor progression and development of metastatic PCa (n=225) from patients with indolent disease (n=367) based on 10-year follow-up data. The authors found that the amount of M2 macrophages and Tregs correlate to each other and that patients with high macrophage numbers (>25 cells within the core) had a 2.05-fold higher risk to progress into lethal disease (281). They conclude that Treg and M2 macrophages have a dominating role to turn the local prostate tumor microenvironment into an immunosuppressive and tumor promoting milieu. Another study published by Di Mitri et al. investigated the same in an experimental PTEN-null prostate-specific conditional (pc−/−) mouse model and identified the CXCL1/CXCL2/CXCL5-CXCR2 signaling as major driver to polarize TAMs into CD45^+^CD11b^+^LY6G^−^F4/80^+^ macrophages with M2 phenotype. Moreover, they found that CXCR2 blockade leads to TAM re-education into M1, tumor regression, increased T cell response, and decreased vessel size. The TAM reprogramming was associated with increased TNFα secretion and induction of senescence in PCa (282). Macrophages within the bone, so-called osteal macrophages, are located adjacent to osteoclasts and regulate bone formation and skeletal homeostasis under physiological conditions. Metastasis-associated macrophages (MAMs) within metastatic PCa lesions are actively recruited *via* IL-6 secreted by PCa cells and promote bone metastasis formation (283). Another immune regulator responsible for DTC immune evasion is the high TGF-β concentration within bone metastasis that is released either through bone matrix remodeling or secreted by osteoblasts. TGF-β induces polarization of CD4^+^ T helper into Th17 and Treg lineage and restrains Th1 cells (284). Jiao et al. hypothesize that this mechanism is the key factor that explains the lack of clinical efficiency of immunotherapies in metastatic CRPC patients and indicates the potential of immune checkpoint therapy in combination with TGF-β inhibitors (285, 286). A recently published study demonstrated that the immunosuppressive microenvironment within PCa bone metastasis can be targeted *via* the CCL20-CCR6 axis. Treatment of mice with syngeneic prostate bone metastases with a CCL20-blocking antibody led to T cell exhaustion and significantly prolonged survival (287). However, further studies are needed to understand the role of immune cell induced and/or regulated DTC dormancy to prevent rapid interruption, re-activation, mobilization and further metastatic progression of novel targeting agents. Another highly interesting research focus with therapeutic potential are investigations of immune signals from the primary tumor to form a pre-metastatic, “primed” niche at a distant site.

## Conclusion

Elucidation of the molecular and cellular mechanisms that drive tumor cell dissemination and regulate cellular response to radiotherapy is essential for developing novel diagnostic criteria and individualized therapeutic strategies. Today, systemic therapy remains standard of care, even in patients with no or up to three visible metastases. However, PCa patients may benefit from metastasis-directed therapy, e.g., based on stereotactic ablative radiotherapy, in combination with immediate androgen deprivation or extension of systemic therapy. Moreover, PCa patients with oligo-metastatic disease are a heterogeneous subgroup of patients and urgently need a better stratification system to improve standard of care. Blood-based biomarkers such as circulating tumor cells (CTCs) are a unique non-invasive method with enormous clinical utility for patient stratification and monitoring in particular for patients with metastatic disease.

## Author Contributions

All authors contributed to the article and approved the submitted version.

## Funding

DK and CP are supported by the Deutsche Forschungsgemeinschaft (DFG) and the priority program 2084 “µBONE: Colonization and interaction of tumor cells within the bone microenvironment” (project number 401326337).

## Conflict of Interest

The authors declare that the research was conducted in the absence of any commercial or financial relationships that could be construed as a potential conflict of interest.
